# On the Development of a Sense and Avoid System for Small Fixed-Wing UAV

**DOI:** 10.3390/s25082460

**Published:** 2025-04-14

**Authors:** Bruno M. B. Pedro, André C. Marta

**Affiliations:** IDMEC, Instituto Superior Técnico, Universidade de Lisboa, 1049-001 Lisboa, Portugal; bruno.pedro@tecnico.ulisboa.pt

**Keywords:** obstacle detection, collision avoidance, vector field histogram, flight controller, companion computer, ultrasonic sensor, laser rangefinder, LiDAR

## Abstract

The increasing use of Unmanned Aerial Vehicles (UAVs) demands enhanced flight safety systems. This study presents the development of an affordable and efficient Sense and Avoid (S&A) system for small fixed-wing UAVs, typically under 25 kg and fly at speeds of up to 15 m/s. The system integrates multiple non-cooperative sensors, two ultrasonic sensors, two laser rangefinders, and one LiDAR, along with a Pixhawk 6X flight controller and a Raspberry Pi CM4 companion computer. A collision avoidance algorithm utilizing the Vector Field Histogram method was implemented to process sensor data and generate real-time trajectory corrections. The system was validated through experiments using a ground rover, demonstrating successful obstacle detection and avoidance with real-time trajectory updates at 10 Hz.

## 1. Introduction

Unmanned Aerial Vehicles (UAVs) have evolved from primarily military applications to a wide range of civil and commercial uses, such as in surveillance, agriculture, logistics, and media [1]. These applications often require UAVs to operate at low altitudes, where obstacles like buildings, trees, and power lines pose significant collision risks. Consequently, the rapid expansion of the UAV market [2] emphasizes the need for reliable safety systems.

While significant effort is being put into the popular multirotor platforms, small fixed-wing UAVs are often overlooked in terms of safety systems. To mitigate this, the present work addresses the safety enhancement of fixed-wing UAVs, with Maximum Take-Off Weight (MTOW) under 25 kg and cruise speed in the order of 15 m/s. The focus is on developing a Sense and Avoid (S&A) system aimed at detecting obstacles and avoiding collisions autonomously during flight. These target fixed-wing aircraft applications are characterized by specific dynamics, in particular relatively fast forward flight and limited maneuvering capabilities, which required S&A solutions distinct from those of multicopters.

Obstacle sensing systems in UAVs are generally divided into cooperative detection—when information is exchanged between the aircraft and the obstacle (usually another aircraft), as in Traffic Alert and Collision Avoidance System (TCAS) [3,4] or in Automatic Dependent Surveillance–Broadcast (ADS-B) systems [5,6]—and non-cooperative detection. During the operation of small UAVs, the most common obstacles in the surrounding environment are man-made structures (buildings, bridges, or power lines) and orography (steep terrain or cliffs). For this reason, non-cooperative S&A solutions should be sought for small fixed-wing UAVs, not only for their reduced cost but also for obstacles that do not broadcast their position [7].

Non-cooperative obstacle sensing requires proper hardware. Active sensors determine the distance to an object by measuring the time lapse between the emission of a wave and the reception of its reflection from the object. Examples include Radio Detection and Ranging (RADAR), laser rangefinders, and Light Detection and Ranging (LiDAR) sensors, which use electromagnetic waves, as well as ultrasonic sensors (sonars), which use sound waves. Laser rangefinders are potentially more accurate than RADARs and ultrasonic sensors due to the usage of electromagnetic waves with shorter wavelengths, which allow for higher spatial resolution. Passive sensors rely on capturing energy emitted by external sources. The most common are electro-optical sensors, such as those in video cameras, which consist of an array of pixels capable of measuring the intensity of electromagnetic waves in the visible wavelength range. The captured images must be processed based on visual features, such as edge, color, size, texture, shape, and optical flow, to detect the obstacles and estimate their position and motion.

The application of body-fixed laser rangefinders for obstacle detection and avoidance in a quadrotor UAV was simulated in [8], assessing the feasibility of different configurations regarding the number of sensors and installation angles. LiDAR, characterized by allowing wider scanning angles, was considered, for instance, in [9] for small fixed-wing UAVs, and it was simulated as part of an S&A system. It proved to be a good option for obstacle detection and allows for the further generation of optimized trajectories to safely avoid obstacles in a wide range of weather and geometric conditions. The ultrasonic sensors are a cheaper and lighter option for obstacle detection. However, they have a limited detection range compared to previous sensors, such that they are usually implemented in combination with other sensors. In [10], a low-cost obstacle detection and collision avoidance solution for quadrotors is proposed, which uses data fusion from twelve ultrasonic sensors and sixteen infrared sensors for 360° coverage. The electro-optical sensors require real-time image processing, which makes them of limited application in low computational power airborne platforms [11]. Nevertheless, the optical flow technique has been successfully demonstrated in multicopters [12], where motion parallax is used to calculate the displacement of pixels between consecutive image frames and identify the relative motion between the camera and the obstacles.

Collision avoidance often requires the aircraft to adjust its flight path in order to perform an evasive maneuver. Path planning methods for collision avoidance in UAVs can be global when the obstacles are known before a flight or local when the obstacles are not expected, and the path is updated in real time. Among the global methods, variations of the graph-based algorithms A* [13] and Rapidly exploring Random Tree (RRT) [14] are the most commonly found in UAV applications [15,16,17]. In the case of local methods, which are the most important in this work, it is common to find the following: (i) geometric methods, which generate paths to avoid collisions with obstacles by taking advantage of their geometry, relative position, and relative velocity [18,19,20]; (ii) potential field methods, which model the space around a UAV, creating a field of attractive and repulsive forces to guide a UAV to avoid the obstacles [21,22]; (iii) gap-based methods, which search for a path to move a UAV through the most suitable space gap between obstacles in the environment. Of the latter, it is worth noting the Vector Field Histogram (VFH) method [23], which represents the environment around the UAV in a polar histogram used for selecting the direction with less density of obstacles. In [24], a slightly modified version of the VFH was successfully validated in real flight tests with seven similar fixed-wing UAVs, using onboard host computers running Ubuntu 14 and Pixhawk flight controllers running a feed-forward PID control algorithm.

When designing an S&A system for fixed-wing UAVs, which cannot hover, the system’s response time is critical for a safe avoidance maneuver. This time is affected by the update rate of the sensors, the computational delay of data processing, and the latency of the actuator commands. The speed and maneuverability of the UAVs are essential to determine the limit of the timeframe within which this response must fall. Although a detailed approach to UAV maneuvering is left out of this study, the response time of the S&A system is explored concerning UAV speed and sensor detection ranges.

Building upon previous works that modeled specific sensors and optimized sensing configurations for a small fixed-wing UAV [25,26,27], this study presents a novel S&A system integrating multiple non-cooperative sensors and a real-time collision avoidance algorithm. The key contributions are as follows:

- The design and implementation of a complete hardware solution for the system, which incorporates a sensor configuration with two ultrasonic sensors, two laser rangefinders, one LiDAR, a flight controller, and a companion computer;
- The implementation of a software solution for the system based on the adaptation of the open-source flight control software, PX4, and the development of a software prototype to process sensor data, which compute obstacle positions and apply the VFH method for collision avoidance trajectory re-planning in real time;
- The experimental validation of the overall S&A system through bench testing in a ground rover robot, which provides a foundation for future UAV flight tests.

## 2. S&A Hardware Implementation

The hardware implementation of an S&A system requires some decisions regarding the physical components that comprise it, as well as how they are configured and connected.

### 2.1. Range Sensors

The key components of the sensing system are the range sensors, which should provide accurate distance measurements between the aircraft and the surrounding obstacles.

The optimization study performed in [27,28] compared different sensing configurations using ultrasonic sensors, laser rangefinders, LiDAR, and RADAR sensors in a simulation environment. It concluded that the best configurations consist of a front-facing LIDAR accompanied by either two laser rangefinders pointing sideways at ±10° or two RADARs at ±28°. From these, the first configuration was chosen, given the lower cost of the laser rangefinders compared to RADARs. Moreover, the ultrasonic sensors were not discarded, given their potential to be used, for instance, in low-speed ground operations covering blind spots of the other sensors.

**Figure 1:**
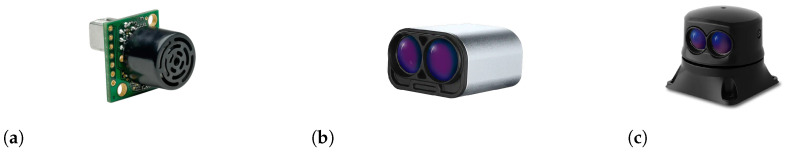
Range sensor hardware components: (**a**) Ultrasonic sensor Maxbotix MB1242 [29], (**b**) Laser rangefinder Lightware LW20/C [30], (**c**) LiDAR Lightware SF45/B [31].

In summary, the hardware chosen to support the obstacle detection is composed of three different types of non-cooperative active sensors: two ultrasonic sensors (Figure 1a), two laser rangefinders (Figure 1b), and one LiDAR (Figure 1c). The update rate of the sensors has a major impact on the overall response time of the S&A system.

Two different models of ultrasonic sensors from the Maxbotix are used, MB1202 and MB1242, which share specifications, such as detection range up to 7.65 m, resolution of 1 cm, accuracy of 10 cm, and maximum update rate of 10 Hz constrained by the duration of a ranging cycle. The major difference between them is the type of beam pattern, which is wider for MB1202 (more noise clutter) and narrower for MB1242 (less noise clutter). To operate both sensors simultaneously on the same bus of the Inter-Integrated Circuit (I2C) communication interface and distinguish their range measurements, they must have different addresses Thus, the I2C address of MB1202 was changed to 0x68 and MB1242 to 0x70.

Two identical laser rangefinders, Lightware LW20/c, are considered. Their detection range goes up to 100 m, much higher than that of the ultrasonic sensors. Since they rely on the speed of light instead of the speed of sound, the update rate can also be much higher. Moreover, they are tolerant to changes in background lighting conditions, wind, and noise. The accuracy is not generally affected by the color or texture of the target surface, nor the angle of incidence of the beam [30], as opposed to the ultrasonic sensors. Regarding the communication interface, it was chosen to use I2C; therefore, the I2C address of one laser was changed to 0x67 using the Lightware Studio software provided by the manufacturer, and the other kept the original 0x66.

To scan a wider area ahead of the UAV, the Lightware SF45/B LiDAR sensor is considered. With a detection range up to 50 m, the major features of this LiDAR are the scanning angle, which can be set from 20° to 320°, and the update rate, configurable from 50 Hz to 5000 Hz. The speed of rotation depends on the scan angle and can go up to 5 sweeps per second. Similarly to the laser rangefinder, it is also tolerant to changes in background lighting conditions, wind, and noise [31]. The scanning angle was configured to range from −45° to 45°, given the turning rate limitations of a fixed-wing UAV. In this case, it was chosen to use serial over I2C as a communication interface through one of the telemetry (TELEM) ports of the flight controller.

The main specifications of the selected sensors are summarized in Table 1.

### 2.2. Flight Controller and Companion Computer

The obstacle detection sensors are connected to the flight controller, which collects and processes their measurements in the first instance. Due to the flight controller’s limited computational power, the processing necessary for the application of a collision avoidance method is executed by a more powerful companion computer that directly communicates with it.

**Figure 2:**
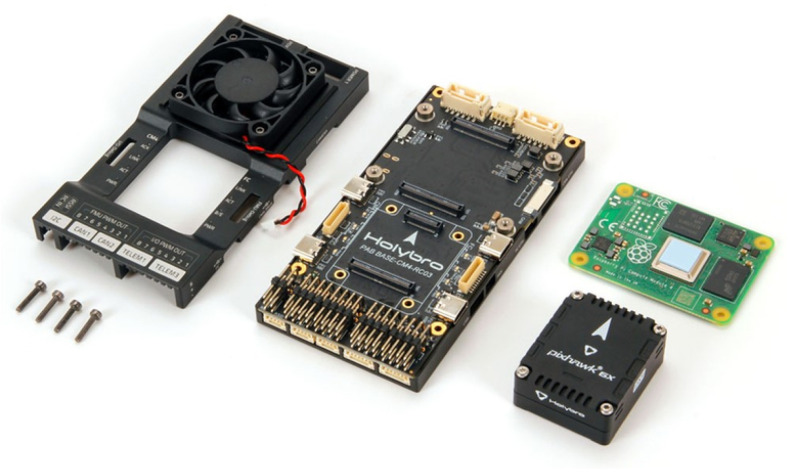
Flight controller Holybro Pixhawk RPi CM4 baseboard parts (from left to right): case with fan, baseboard, Pixhawk 6X, and Raspberry PI CM4 [32].

The flight controller chosen for this application is the Pixhawk 6X from Holybro, which, together with the Raspberry Pi Computer Module 4 (CM4) as a companion computer, is integrated into the Holybro Pixhawk RPi CM4 baseboard [32], as presented in Figure 2.

### 2.3. Electrical Layout

**Figure 3:**
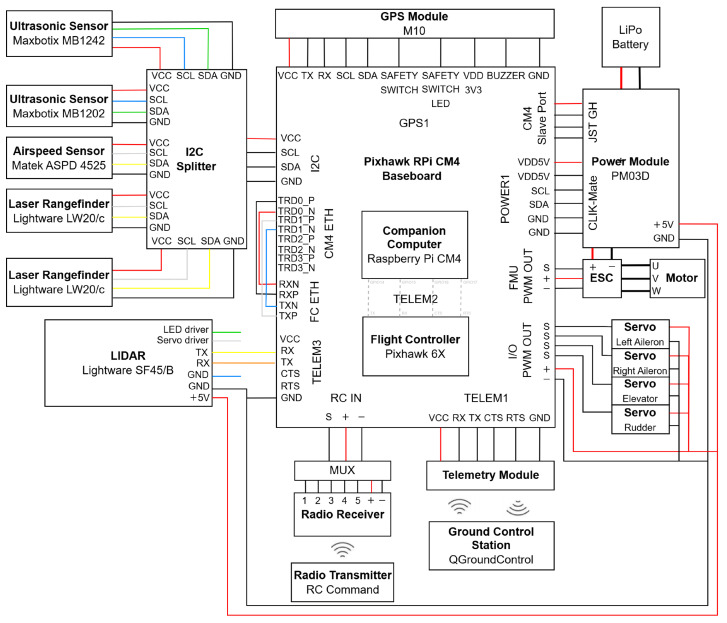
UAV flight control and S&A systems: hardware electrical diagram.

The electrical layout of the hardware connections is shown in Figure 3, including the auxiliary components essential for the flight operation of a fixed-wing UAV, such as power module, battery, electric motor, servos, Electronic Speed Controller (ESC), Global Positioning System (GPS) module, radio receiver and multiplexer (MUX), and telemetry module.

Even though the companion computer and the flight controller are internally connected in the baseboard through the serial TELEM2 port, an Ethernet connection was used instead due to its higher bandwidth.

## 3. S&A Software Implementation

**Figure 4:**
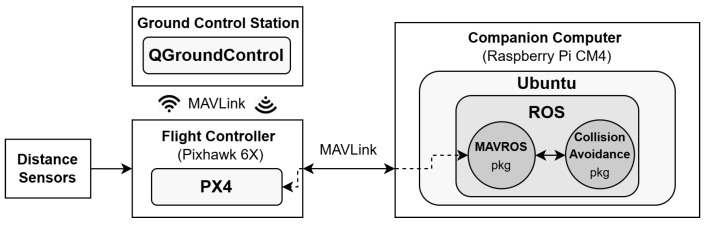
S&A system software: implementation diagram.

The software implementation of the S&A system addressed in this work can be seen as an application with additional developments of existing open-source solutions. Figure 4 presents the diagram of the software’s main components, namely flight control software, ground control software, and companion computer software, and the high-level interaction between them.

### 3.1. Flight Control Software

The flight control software adopted is the PX4 open-source project [33] due to its reliability, modular architecture, allowing for extension of functionalities, good documentation, and increasing presence in the industry, with a growing community of users and developers. It supports different types of vehicles, such as multicopters, fixed-wing UAVs, and rovers.

#### 3.1.1. Internal Communication

The communication between internal modules of PX4 is performed using the micro Object Request Broker (uORB) protocol, which is based on a mechanism to publish/subscribe messages in topics, allowing multiple independent instances of the same topic. Each uORB topic must have a prior definition of the fields that make up its message context.

The data from the obstacle detection sensors are published in the distance_sensor uORB topic, whose fields are described in Table 2. The most important fields are the device_id, a unique ID of the sensor, the current_distance, the sensor range measurement, and the current_yaw, which is the only non-standard field, added to include the direction of the LiDAR in the horizontal plane, in degrees.

The goal is to have the sensor data published in a single distance_sensor uORB topic, with one instance for each sensor. However, the PX4 driver (Section 3.1.2) of the ultrasonic sensors is prepared for processing data from multiple sensors, so the two ultrasonic sensors end up sharing the same instance of the uORB topic. Consequently, all sensor data are internally organized in four different instances of the same distance_sensor uORB topic to, then, be streamed over MAVLink (Section 3.1.3) to both the Ground Control Station (GCS) and the companion computer.

Other uORB topics are also used in the S&A system. For example, the following is true: (i) vehicle_local_position is used to communicate the UAV local position, velocity and acceleration estimates in a NED (North-East-Down) frame; (ii) trajectory_setpoint is used to internally communicate position, velocity and acceleration setpoints in a local NED frame; (iii) vehicle_local_position_setpoint can be used to monitor the setpoints inputted to the position controller of PX4.

#### 3.1.2. Distance Sensor Drivers

The interface between obstacle detection sensors and the PX4 is made possible by drivers, which are responsible for sensor initialization, acquisition of data measurements, primary data processing, and communication with the uORB messaging bus. These drivers can be controlled through MAVLink Console commands.

Ultrasonic sensors are controlled by the built-in mb12xx PX4 driver, whose single instance can control multiple ultrasonic sensors connected to the same I2C bus, provided they have different I2C addresses. The sensor update rate is defined by its driver to 10 Hz to match the maximum ranging cycle time of around 100 ms, and since the same driver controls two sensors, a 50 ms interval is set between consecutive sensor reads to meet the ranging cycle requirement per sensor.

The laser rangefinders are controlled by the built-in lightware_laser_i2c PX4 driver. Contrary to what happens with the ultrasonic sensors, this driver is unable to control, in a single instance, multiple sensors with different I2C addresses in the same I2C bus. For this reason, the solution found to have two lasers connected at the same time was to start two independent instances of the driver in the startup shell script of PX4.

The LiDAR is controlled by the built-in lightware_sf45_serial PX4 driver. Unlike the previous drivers, it is not included in the firmware by default, so it needs to be manually enabled in the PX4 firmware configuration. Although the LiDAR sensor measures the scanning angle at each instant, the standard version of its driver does not publish these measurements, which are necessary to fulfill the custom current_yaw field added to the distance_sensor uORB topic. So, for this purpose, the s_update() function developed in [27] was included.

#### 3.1.3. External Communication

External communication between the flight controller and other devices, such as the GCS and the companion computer, is performed through Micro Air Vehicle Link (MAVLink). MAVLink messages are characterized by a name, an id, and fields containing the data to be transmitted. PX4 includes MAVLink as a module, and generally, MAVLink messages stream data of an already existing uORB message with similar fields. Furthermore, it can have independent instances to communicate with different peripheral devices simultaneously.

DISTANCE_SENSOR (ID=132) is the standard MAVLink message used to communicate data from the obstacle detection sensors. Although most of its fields are similar to those of the homonym uORB topic, it had to be slightly modified to suit the S&A system, with the custom addition of the device_id and current_yaw fields, to hold the unique sensor identifier and LiDAR yaw angular position (when applicable), respectively.

Among many other standard MAVLink messages, the ones most relevant to the S&A system are the following: (i) LOCAL_POSITION_NED, used to communicate the UAV local position; (ii) SET_POSITION_TARGET_LOCAL_NED, used to communicate position, velocity or acceleration setpoints defined by the collision avoidance algorithm running in the companion computer; (iii) POSITION_TARGET_LOCAL_NED, used to retrieve data from the vehicle_local_position_setpoint uORB topic to monitor the setpoints that are actually being sent to the position controller of PX4; (iv) VFR_HUD, used to communicate head-up display (HUD) information, such as airspeed, ground speed, heading, throttle, altitude MSL, and climb rate.

Since, in addition to the previous ones, a few more standard messages are communicated over MAVLink, the streaming rate of data is conditioned by the flight controller’s processing power, the congestion of the link, as well as the characteristics of the physical connection.

### 3.2. Ground Control Station Software

The GCS is a ground-based system that allows a human operator to monitor, control, and manage the systems of a UAV in real time. The open-source QGroundControl [34] was used as the GCS software due to its proven integration with PX4. It ran on a computer with Windows 11 OS to communicate with PX4 over USB (wired) or telemetry (wireless).

The telemetry module responsible for the physical connection between the flight controller and the GCS limits, significantly, the streaming rate of the MAVLink messages received by QGroundControl.

### 3.3. Companion Computer Software

A communication link between the companion computer and the flight controller is needed. It is used to exchange data, namely for the companion computer to receive the obstacle detection sensors data from the flight controller and to send it new setpoints to perform the collision avoidance maneuver.

Firstly, an Ethernet connection between them is set, since it has a much higher bandwidth compared to serial connections and can handle high streaming rates of data. Then, the MAVLink interface to use in the companion computer was chosen among MAVSDK, pymavlink, and MAVROS. MAVSDK [35] is a cross-platform high-level API to interface with MAVLink, that is easy to use, but it has limited low-level access and control over the messages. In contrast, pymavlink [36] is a low-level Python library that provides fine-grained control of the MAVLink messages, but it presents a steeper learning curve. Lastly, MAVROS [37] is a Robot Operating System (ROS) package that acts as a bridge between ROS and MAVLink by translating MAVLink messages to/from ROS messages, organized in ROS topics. Since it includes well-tested PX4 support and allows the integration of the S&A system as a ROS package, MAVROS 1.19.1 with ROS1 Noectic was the option selected to interface with MAVLink despite being more resource-intensive.

To communicate distinguishable data from the five obstacle detection sensors, the distance_sensor plugin of MAVROS had to be modified to map the sensors from the device_id field. Moreover, a custom ROS message was created to include the device_id and current_yaw fields.

Although the physical Ethernet connection does not represent a constraint for the streaming rate of data, the MAVLink streaming rate is limited to 20 Hz to avoid a link overload due to the large number of MAVLink messages that are communicated to the companion computer. Therefore, even though the update rate of the laser rangefinder and the LiDAR are higher, the streaming of their reads to MAVROS is limited to 20 Hz. The ultrasonic sensor’s reads are not affected because they are limited to 10 Hz by hardware.

### 3.4. Collision Avoidance Software

Given that ROS provides a flexible framework for writing robotic software with MAVROS as a MAVLink interface, the remaining steps of obstacle detection and collision avoidance can be developed as a software prototype within a ROS package. This approach is not a novelty since there is already an open-source package developed by the PX4 community, PX4-Avoidance [38], to enable obstacle detection with a stereo-vision camera hardware and collision avoidance for multicopters.

Regarding the programming language, Python was chosen in this stage of development for rapid prototyping, although C++ allows better performance. The rospy Python library provides an interface with ROS for the creation of nodes, publish/subscription of topics, and interaction with services and parameters. A multithreading approach was considered, with the threading Python module, to allow multiple tasks to run concurrently within a single process.

The software prototype was divided into two main parts: (1) obstacle detection, which is responsible for processing the data from the distance sensors and transforming it into two-dimensional positions; (2) collision avoidance, which is responsible for generating, in real time, an avoidance trajectory for the UAV. The approach followed here was based on the VFH method [23,24].

#### 3.4.1. System Architecture

**Figure 5:**
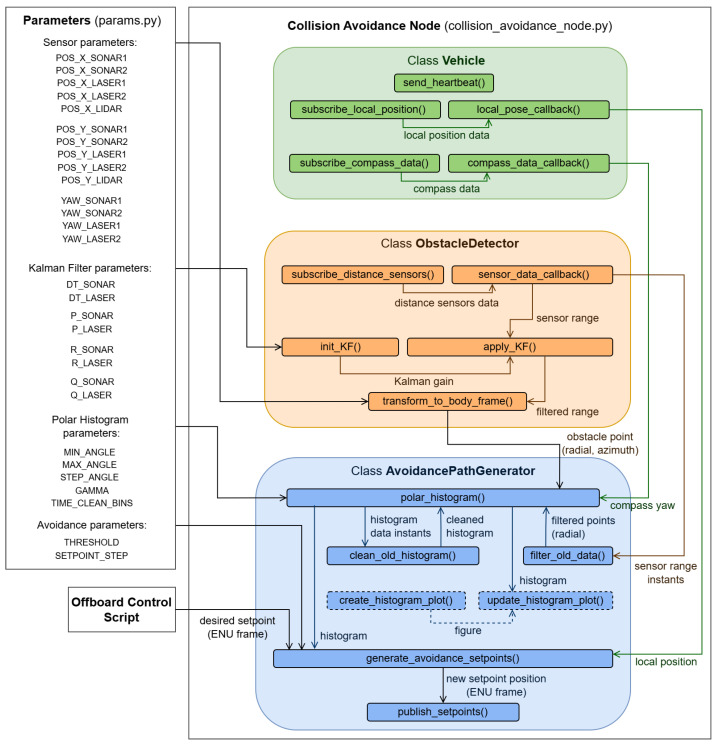
Obstacle detection and collision avoidance system architecture.

The architecture of the S&A system software prototype is illustrated in Figure 5 and includes the files, classes, methods, and the data flow between methods. It is organized in two main files: Parameters (params.py), where the main parameters of the system, related to the distance sensors, Kalman filter, polar histogram, and avoidance process are configured/tuned; and the Collision Avoidance Node (collision_avoidance_node.py), where all the code developments are included. The developed source code can be found in [39].

#### 3.4.2. Obstacle Detection Implementation

The obstacle detection part of the software is implemented within the class **ObstacleDetector**. It starts with the subscription of five ROS topics, one for each sensor, where data are being published by MAVROS. This way, every time new sensor data are published on the corresponding topic, a callback function is called to save it in sensor-specific variables and process it. A one-dimensional Kalman filter, from the filterpy Python package, is applied to the range measurements of the ultrasonic sensors and laser rangefinders that are within the detection range considered (Table 3) to smooth noisy sensor data and provide a better estimate $\hat{d}$ of the true distance to the obstacles.

Then, the filtered range measurements of the sensors $\hat{d}$ are transformed to polar coordinates in the UAV body reference frame to represent two-dimensional obstacle positions. For this, the position in Cartesian coordinates $\left(x_{sens},y_{sens}\right)$ and orientation $\beta_{sens}$ in the body frame, specified as parameters for each sensor installed on the UAV, are used to compute the radial and azimuthal components of the obstacle position, $\left(r_{obs},\varphi_{obs}\right)$, from

(1) $$r_{obs}=\sqrt{{\left(\hat{d}\cos\left(\beta_{sens}\right)+x_{sens}\right)}^{2}+{\left(\hat{d}\sin\left(\beta_{sens}\right)+y_{sens}\right)}^{2}}$$

and

(2) $$\varphi_{obs}=\arctan\left(\frac{\hat{d}\sin\left(\beta_{sens}\right)+y_{sens}}{\hat{d}\cos\left(\beta_{sens}\right)+x_{sens}}\right)\;.$$

#### 3.4.3. Collision Avoidance Implementation

**Figure 6:**
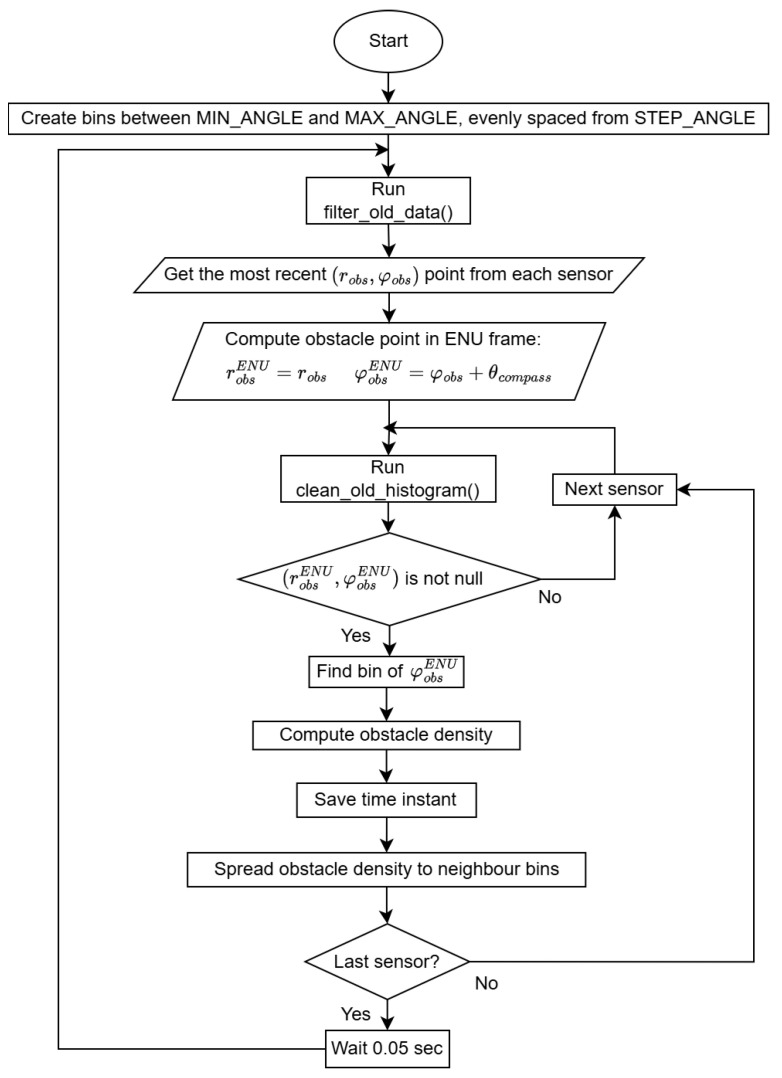
Polar histogram creation and continuous update algorithm.

The collision avoidance part of the software is implemented mainly within the class **AvoidancePathGenerator**, using some methods of the class **Vehicle**. Having the position of the obstacles detected by the sensors in polar coordinates of the body frame and aiming to apply the VFH method, a polar histogram is generated to represent the obstacle density in space using the algorithm flowchart in Figure 6.

It starts with the creation of the polar histogram using the parameters MIN_ANGLE and MAX_ANGLE to define the angular range of space coverage around the UAV, and STEP_ANGLE to define the resolution of the histogram, i.e., the angular step covered by each bin. Then, there is a loop with a frequency of 20 Hz, which is the expected streaming rate of MAVLink messages with new sensor reads from the laser rangefinders and the LiDAR, to update the obstacle density value corresponding to each histogram bin using the most recent obstacle position detected by each sensor. The obstacle positions, $\left(r_{obs},\varphi_{obs}^{ENU}\right)$, in East-North-Up (ENU) frame, are used to find the corresponding bin *k* of the histogram, in which the obstacle is inserted, and compute the obstacle density, $h_{k}$, using the arbitrary function

(3) $$h_{k}=\frac{50-r_{obs}}{50}\;,$$

ensuring obstacle significance decreases linearly with distance. The constant of 50 was chosen to correspond to the maximum detectable distance considered for the laser rangefinders and the LiDAR, thus normalizing the bin obstacle density value. For safety reasons, the obstacle density of a bin is spread to its neighbor bins, using a function controlled by the parameter γ as

(4) $$h_{k\pm a}=h_{k},\;a=1,\cdots ,\gamma \;.$$

The continuous update process includes methods to erase old sensor data as well as old histogram data.

**Figure 7:**
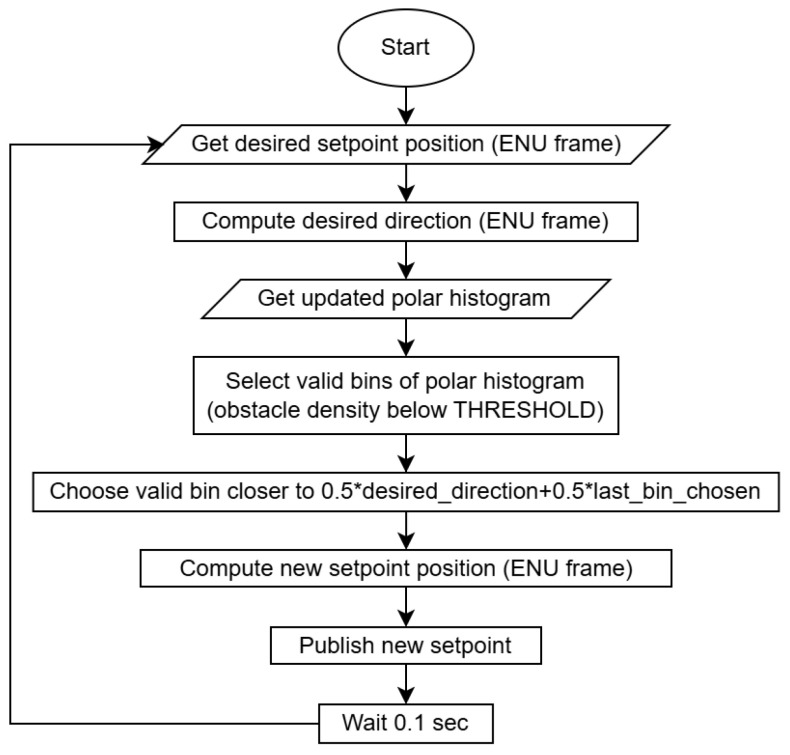
Avoidance setpoints generation algorithm.

Concurrently to the update of the polar histogram, new trajectory setpoints for the UAV are generated with a frequency of approximately 10 Hz from the algorithm flowchart of Figure 7, in order to wait for new data from all sensors, including the hardware limited 10 Hz update rate of the ultrasonic sensors.

This process starts from desired setpoint positions in ENU frame, which are given by an external off-board control script, and are used to determine the desired direction.

Then, the bins of the polar histogram with an obstacle density below a prescribed THRESHOLD (available bins) are selected and, from these, the one corresponding to a direction closer to the average between the desired direction and the direction followed in the last iteration is chosen. From that direction, a new setpoint velocity in Cartesian coordinates is generated using the SETPOINT_STEP parameter, as well as a new setpoint position in the ENU frame using local position data. Finally, the new setpoint position or velocity can be published.

## 4. S&A System Validation Tests

To validate the proposed S&A system hardware and software architectures and corresponding implementations, a few real-world tests were conducted. Given the risks associated with testing these new developments in a fixed-wing UAV in flight, a small unmanned ground vehicle (UGV), hereinafter referred to as a rover, was used instead at this stage. The following sections present the S&A system hardware and software setup in the rover, as well as three basic tests performed: (i) static vehicle and multiple static obstacles; (ii) static vehicle and a moving obstacle; (iii) moving vehicle and a static obstacle.

### 4.1. Rover System Setup

**Figure 8:**
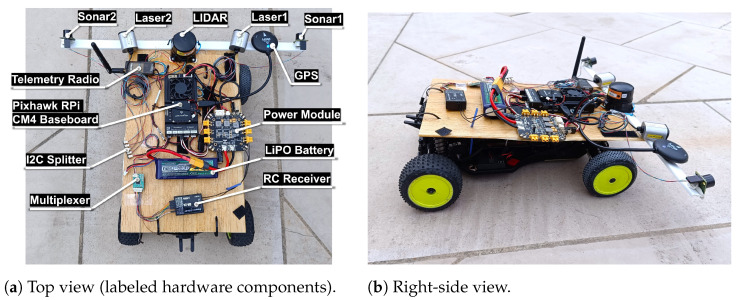
Rover setup for S&A system validation.

The hardware for testing in a rover was adapted from the electrical layout in Figure 3, resulting in the setup in Figure 8. For flight control software, the rover_pos_control module of PX4 1.14.3 was used.

The positioning of the laser rangefinders and the LiDAR in the vehicle frame reproduced the optimal configuration presented in [28]. The ultrasonic sensors were placed facing forward in the most distant lateral points to cover the blind spots of the other sensors at low distances.

### 4.2. Static Vehicle and Static Obstacles

**Figure 9:**
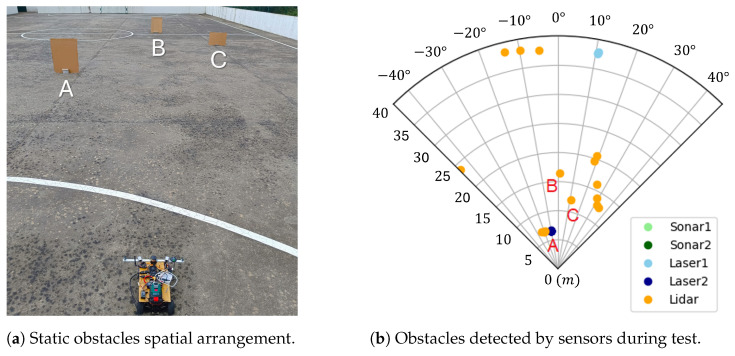
S&A system test with static vehicle and static obstacles.

The first test was conducted with the vehicle static in front of three obstacles, which were also static, of different frontal areas, A (0.55 m^2^), B (0.55 m^2^) and C (0.35 m^2^), which were arranged as shown in Figure 9a. The goal was to validate the capabilities of the S&A system to detect obstacles, estimate their relative positions, and translate them to the polar histogram of the VFH method. The system ran for around 10 s.

Table 4 presents the parameters used for sensors, Kalman filters, polar histogram, and avoidance algorithm. The sensor parameters reflect their two-dimensional position, $\left(x_{sens},y_{sens}\right)$, in the vehicle’s body frame, relative to its estimated center of mass, and their orientation, $\beta_{sens}$, relative to the vertical axis of the vehicle’s body frame. The Kalman filter parameters used resulted from a previous tuning process that aimed to set dt with the respective sampling interval of the ultrasonic sensors and laser rangefinders, *P* with a realistic estimate of the initial sensor measurements, and the process noise covariance, *Q*, and the measurement noise covariance, *R*, with a relative difference of one order of magnitude to allow the filtering of outliers and noise, while keeping the estimations responsive to sudden changes in measurements. The polar histogram parameters were set for it to have a 360° coverage, bins with a width of 10°, and two neighbors each (γ=2). Regarding the avoidance process, the TIME_CLEAN_BINS parameter was set for the histogram to keep its bins for 0.1 s before cleaning, and the THRESHOLD for a normalized obstacle density of 0.8 and the SETPOINT_STEP for the next trajectory setpoint positions were to be placed according to a vector with 3 m of magnitude.

The data of the positions of the obstacles in polar coordinates in the rover body frame, $\left(r_{obs},\varphi_{obs}\right)$, are plotted in Figure 9b and separated by range sensor data source.

It can be observed that the ultrasonic sensors did not detect any obstacles since they were outside their range, the laser rangefinders detected obstacles along the directions they were pointed to, and the LiDAR detected many in different directions, as expected. It is possible to identify obstacle A, which was successfully detected by both Laser2 and LiDAR, as well as obstacles B and C, which were only detected by LiDAR. The cluster of LiDAR points on the right-hand side, the single point on the left-hand side, and the points further away around 40 m distance, including the one from Laser1, correspond to the detection of the walls of the sports field where the test was conducted. From this test, it was possible to conclude that the obstacle sensing system can detect the targets successfully using both the laser rangefinders and LiDAR, and that the sonars are of limited use due to their reduced range.

**Figure 10:**
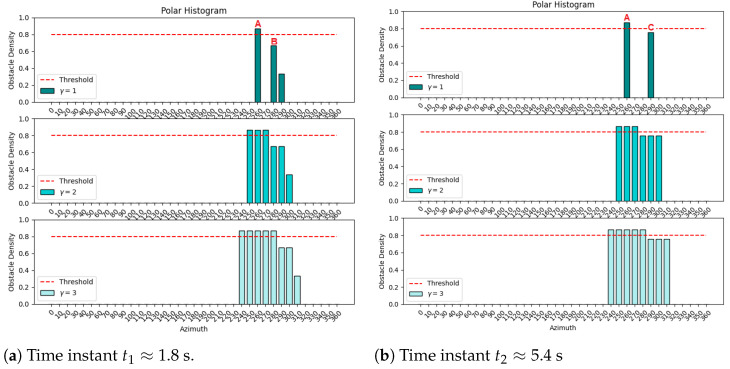
S&A system test with static vehicle and static obstacles (A,B,C): polar histograms for γ=1,2,3 (top to bottom).

The translation of the obstacles’ positions to the polar histogram, with bins from 0° to 360°, and step angle of 10°, were plotted in Figure 10a,b, from two time instants, $t_{1}$ and $t_{2}$, when the pairs of obstacles (A,B) and (A,C) were detected, respectively.

At time t≈1.8 s, there are three main bins, one for 260° from Laser2’s detection of obstacle A, another for 280° from the LiDAR detection of obstacle B, and a smaller bin for 290° from Laser1’s detection of the wall. At time t≈5.4 s, there is the same main bin for 260° and another for 290° from the LiDAR detection of obstacle C. The plots in the second and third rows of Figure 10 present the effect of spreading the obstacle density to neighbor bins, which are controlled by γ as defined in Equation (4). Having γ=1 leads to zero neighbors, γ=2 leads to two neighbors for each main bin, and γ=3 leads to four neighbors for each main bin. This parameter can be used to tune the allowable safety margin around obstacles. A good trade-off needs to be found since a higher γ corresponds to safer, more conservative, obstacle detection at the expense of reducing obstacle-free path alternatives for the collision avoidance algorithm. An example of a threshold line of 0.8 is also presented—the bins with obstacle densities above 0.8 are considered unavailable, while the others are available in the collision avoidance VFH algorithm.

### 4.3. Static Vehicle and Moving Obstacle

The second test was performed with the vehicle static and an obstacle of frontal area 0.55 m^2^ moving in front of it, from left to right, at a speed of about 1 m/s and a distance within an interval between 3 m and 4 m. This interval can be transcribed in the local body frame of the rover as

(5) $$\frac{3}{\cos\theta_{obs}}\leq r_{obs}\leq \frac{4}{\cos\theta_{obs}},\;-45^{{}^{\circ}}\leq \theta_{obs}\leq 45^{{}^{\circ}}.$$

The objective of this test was to validate the detection of dynamic obstacles and their reflection in variations of the polar histogram. The same parameters in Table 4 were used, except for THRESHOLD, which was changed to 0.9 to make the system less sensitive and react only to obstacles with radial distance under 5 m from the vehicle.

**Figure 11:**
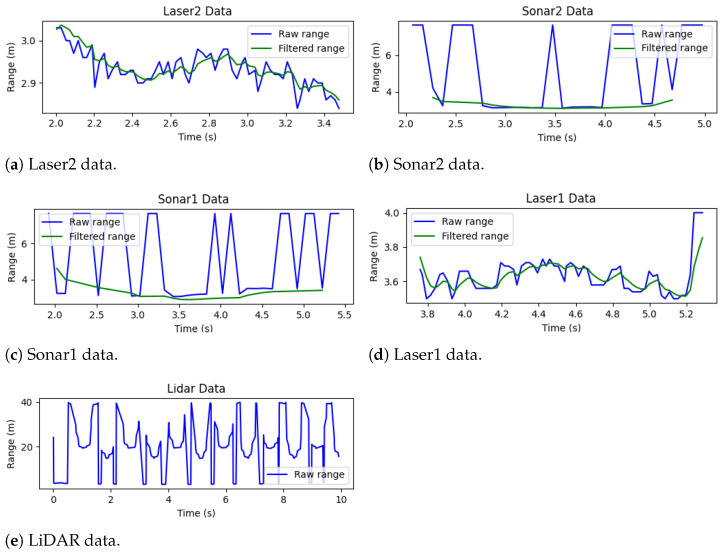
S&A system test with static vehicle and moving obstacle: raw and filtered obstacle detection sensor data.

To assess the characteristics of the sensor data that feed the system, the raw and filtered range measurements of the sensors were saved and truncated to the time intervals when the obstacle is detected. These data are plotted in Figure 11a–e, for each of the five sensors, ordered from the first to the last that detected the obstacle. Given the positions of the sensors in the vehicle, the sequence of detection by the lasers and ultrasonic sensors is as expected for an obstacle moving from left to right.

For all cases, the obstacle was detected through range measurements between 2.8 m and 4 m. Moreover, the Kalman filter performed reasonably for the lasers and ultrasonic sensors, reducing the noise and dampening the effect of outlier measurements that could lead the system to unnecessary reactions. Measurements of the ultrasonic sensors above 7 m were not considered for filtering since these sensors report their maximum range (7.65 m) when no obstacles are detected. Finally, the LiDAR data were not subject to any filtering process but presented good results by detecting the obstacle at each scan.

**Figure 12:**
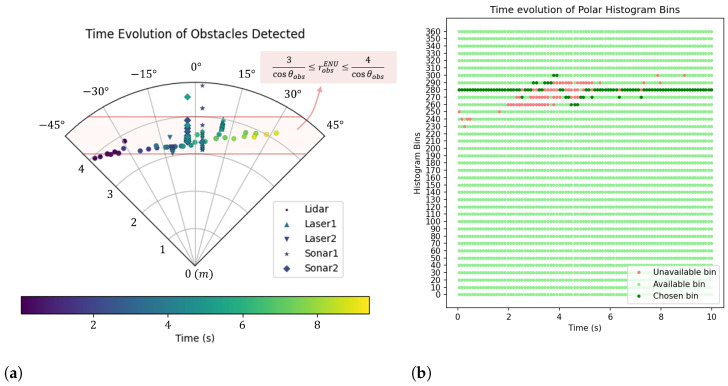
S&A system test with static vehicle and moving obstacle: time evolution of obstacle detection. (**a**) Detected obstacles position in polar coordinates of the vehicle’s body frame. (**b**) Available, unavailable, and chosen bins in polar histogram.

The transformation of the data from all sensors to obstacle positions in polar coordinates in the vehicle’s body frame over the execution of the test resulted in Figure 12a. The points are labeled by the sensors that originated them, such that the cluster of points distributed approximately along the −11° azimuth came from Laser2, the points along the −3° azimuth came from Sonar2, the points along the 3° azimuth came from Sonar1, the points along the 11° azimuth came from Laser1, and the other scattered points came from LiDAR. Once again, the evolution of the point positions in time is in accordance with the trajectory of the obstacle from left to right, as the moving obstacle is detected successfully by the sensing system.

The obstacle positions detected by the sensors were compared with the expected interval defined by Equation (5). The points that are inside/outside the interval and the respective relative error are presented in Table 5 per sensor.

It can be observed that both ultrasonic sensors presented a relatively low error, explained by the appearance of virtual points from the delay of the filtering process. The laser rangefinders made all the detections inside the expected interval, besides also being subject to filtering. The LiDAR was the one with the highest error, probably due to operational errors in the test execution, given that its detection pattern points to a relatively good detection of the obstacle.

The resulting classification into available/unavailable histogram bins is plotted over time in Figure 12b, together with the bins chosen each time. The setpoints arbitrarily input to the system were such that the desired vehicle direction was 280°. As soon as the obstacle covered that direction, the corresponding bin became unavailable, and the system was forced to choose another bin direction. As the obstacle moved to the right-hand side in time, the bins affected were dynamically blocked and released from lower to higher angles while the system was dynamically choosing the available bin closer to the desired direction. From this test, it can be concluded that the system successfully detects a dynamic obstacle, and it is capable of presenting an intended solution to the collision avoidance algorithm.

### 4.4. Moving Vehicle and Static Obstacle

The last test was performed with the vehicle moving toward one static obstacle (0.55 m^2^). The objective was to validate the capabilities of the system to, based on the detection of obstacles, perform a real-world collision avoidance maneuver. The software was tuned with the parameters of Table 4, except the adjusted settings γ=4 and TIME_CLEAN_BINS=1 to enhance safety. Regarding the avoidance part of the software, it was decided to only publish setpoint positions.

**Figure 13:**
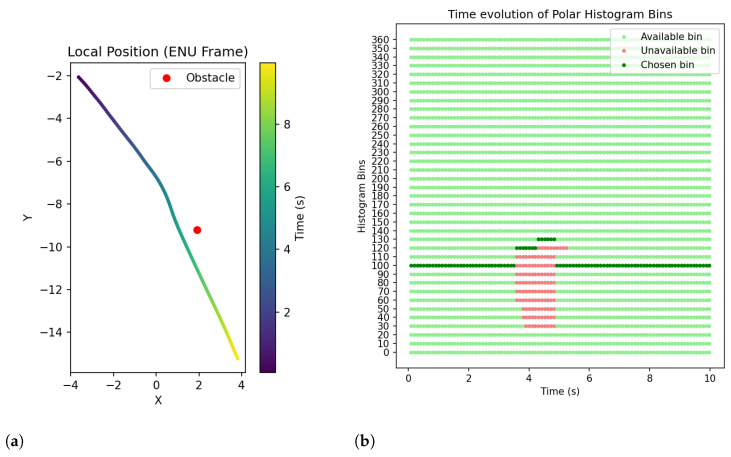
S&A system test with moving vehicle and static obstacle: time evolution of obstacle detection outputs. (**a**) Vehicle local position in ENU frame. (**b**) Available, unavailable, and chosen bins in polar histogram.

The vehicle performed the test at an average speed of 2 m/s. The local vehicle position (in earth-fixed ENU frame) over time is plotted in Figure 13a, together with the position of the obstacle. It shows that the vehicle was following a linear desired trajectory headed toward the obstacle, and around 3 m before the collision, a small trajectory deviation to the right was made, allowing for successful obstacle avoidance.

The classification of histogram bins is plotted in Figure 13b. Initially, the vehicle was physically aligned to the desired heading of 100° and, at the time instant t=3.6 s, the detection of the obstacle led to the blocking of bins from 60° to 110°, forcing the system to choose the hrading of 120° and start the avoidance trajectory. In the following seconds, the blocked bins eventually evolved to the range from 30° to 120°, forcing the vehicle to follow the heading of 130°. When the polar histogram data were cleaned and no further obstacles were detected, the system returned to the desired heading of 100°, finishing the avoidance trajectory.

As shown in Figure 13a, the desired heading followed before starting the avoidance maneuver and after passing the obstacle was not the same, even though the system published setpoint positions in the direction of 100° in both cases. The odd behavior was most likely due to a faulty calibration of the Pixhawk compass, which led to inaccurate heading measures during the test.

The expected rate of trajectory setpoint generation was 10 Hz, without considering the data processing time delay. However, the data analysis of the setpoints published in this test resulted in an average rate of 9.66 Hz, or an average time interval between publishes of 104 ms, meaning an average 4 ms delay in the data processing. Thus, regarding an analysis of the response time of the S&A system, there is a 100 ms contribution to take into account the update of reads from all sensors, added by 4 ms for data processing. The contributions of the communication delay between the sensor measurements and its publishing in the ROS topic, as well as the delay between the publishing of the setpoints in the ROS topic and the actuator commands, were not measured as they were considered small enough to be disregarded.

This demonstrated that the S&A system was able to perform obstacle detection and collision avoidance in the presence of a single obstacle at a vehicle speed of around 2 m/s by generating a trajectory of avoidance setpoint position at a frequency of approximately 10 Hz.

Extrapolating for a fixed-wing UAV flying at 15 m/s, an average response time of the S&A system of 104 ms would delay the start of the avoidance maneuver by 1.56 m, which is reasonably small, considering that the detection range of the laser rangefinder and LiDAR sensors goes up to 50 m.

From the tests performed, it was possible to identify two limitations of the current VFH implementation. First, it was observed that, for the vehicle to choose a direction to follow and perform the avoidance maneuver smoothly without hesitating to follow other mistakenly available bins, there is a need to set a low resolution to the polar histogram. This can be achieved by setting either higher values of the STEP_ANGLE parameter to increase the angular size of each bin or higher values of γ to increase the number of neighbor bins affected by an obstacle detection. This way, the VFH tends to classify narrow gaps as inaccessible, even if they are navigable by the vehicle. Second, the VFH method neglects the dynamics and kinematics of the vehicle under the assumption that it is possible to instantly change the direction of motion at every sampling time. To address these two limitations, future work will explore the improved VFH+ method, which aims to solve the indecisive behavior characteristic of VFH while assuming a more realistic trajectory of the vehicle based on circular arcs and straight lines.

### 4.5. Future Testing Plans

While the rover-based tests provided valuable insights into the performance of the S&A system, they do not fully replicate the aerodynamic and dynamic constraints of a fixed-wing UAV. To address this, future work will include applying the developed system in a simulation environment using the Gazebo open-source robotics simulator, which allows for a smooth integration with ROS and PX4. PX4 supports two different types of flight control simulation: Software In the Loop (SITL) simulation, where the flight stack runs on a computer, and Hardware In the Loop (HITL) simulation, where a simulation firmware runs on an actual flight controller board, as the Pixhawk 6X [33]. The advantages of simulation tests lie in the possibility of testing the S&A system in a risk-free environment using models of sensors, such as Inertial Measurement Unit (IMU), GPS, airspeed sensor, ultrasonic sensors, laser rangefinders, and LiDAR, together with the model of a small fixed-wing UAV which, in turn, is subject to rigid body physics laws, as well as aerodynamic lift, drag, and thrust. These simulations will allow testing of the system in the presence of static and dynamic obstacles of different dimensions and shapes, contributing to the refinement of the avoidance parameters.

After a study of the S&A system behavior in a simulation environment, the conditions are met to proceed to full-scale UAV flight tests using a real fixed-wing UAV equipped with the proposed sensing and avoidance system. These tests will be conducted in restricted airspace, large enough to allow the UAV to reach its cruise speed, and the obstacles will be as collision-safe as possible, such as inflatable air pylons as static obstacles and inflatable air balloons attached to manually controlled multicopters as dynamic obstacles. These tests will allow the validation of the system performance under real flight conditions, focusing on obstacle detection reliability at higher speeds, real-time trajectory correction planning, and safe maneuver execution.

## 5. Conclusions

This work proposed a simple yet efficient S&A system to enhance the flight safety of small fixed-wing UAVs. The system integrates multiple non-cooperative sensors—two ultrasonic sensors, two laser rangefinders, and one LiDAR—along with a Pixhawk 6X flight controller and a Raspberry Pi CM4 companion computer. The software implementation was developed within the PX4 and ROS frameworks, using the Vector Field Histogram method for real-time collision avoidance.

To validate the system, experiments were conducted using a ground rover, demonstrating successful obstacle detection and avoidance. The results confirmed that the system could detect both static and dynamic obstacles, translate them into a polar histogram representation, and generate real-time avoidance maneuvers at approximately 10 Hz.

While promising, these tests were limited to ground-based scenarios. Future work will focus on integrating sensor fusion techniques for robust obstacle tracking and refining the collision avoidance strategy with more advanced methods such as VFH+ or VFH*. Additionally, future system validations will include Gazebo simulations of a fixed-wing UAV model, allowing for controlled and risk-free virtual flight testing before real-world deployment, and, finally, full-scale UAV flight tests to further evaluate system performance in real-world conditions.

## Figures and Tables

**Table 1:** Range sensors’ hardware specifications.

	Ultrasonic Sensor [29]	Laser Rangefinder [30]	LiDAR [31]
Range (m)	0.20–7.65	0.20–100	0.20–50
Scan angle (°)	n/a	n/a	20–320
Resolution (cm)	1	1	1
Angular resolution (°)	n/a	n/a	<0.2
Update rate (Hz)	10	40–388	50–5000
Accuracy (cm)	±10	±10	±10
Power supply voltage (V)	3.3–5	4.5–5.5	4.5–5.5
Power supply current (mA)	2.7–4.4	100	300
Communication interface	I2C	Serial or I2C	Serial or I2C
Dimensions (mm)	22×19×15	30×20×43	51×48×44
Weight (g)	5.9	20	59

**Table 2:** uORB topic distance_sensor fields [33].

Name	Unit	Description
timestamp	ms	Timestamp
device_id	-	Sensor ID
min_distance	m	Minimum range
max_distance	cm	Maximum range
current_distance	cm	Current range
current_yaw *	deg	Current yaw
variance	m^2^	Variance
signal_quality	%	Signal quality
type	-	Sensor type
h_fov	rad	Horizontal Field of View (FOV)
v_fov	rad	Vertical FOV
q	-	Orientation quaternion
orientation	-	Sensor orientation

* non-standard field.

**Table 3:** Detection range values per sensor type for measured data filtering.

Sensor	Detection Range (m)
Ultrasonic sensor	1–7
Laser rangefinder	1–50
LiDAR	1–50

**Table 4:** Obstacle detection and collision avoidance software parameters.

Parameter	Value	Parameter	Value	Parameter	Value
POS_X_SONAR1 (m)	0.2	POS_Y_LASER2 (m)	−0.1	R_LASER	1
POS_Y_SONAR1 (m)	0.2	YAW_LASER2 (°)	−10	Q_LASER	$\left[\begin{array}{cc} 10^{-1} & 0\\ 0 & 10^{-1} \end{array}\right]$
YAW_SONAR1 (°)	0	POS_X_LIDAR (m)	0.2	MIN_ANGLE (°)	0
POS_X_SONAR2 (m)	0.2	POS_Y_LIDAR (m)	0	MAX_ANGLE (°)	360
POS_Y_SONAR2 (m)	−0.2	DT_SONAR	0.1	STEP_ANGLE (°)	10
YAW_SONAR1 (°)	0	P_SONAR	7	GAMMA	2
POS_X_LASER1 (m)	0.2	R_SONAR	1	TIME_CLEAN_BINS (s)	0.1
POS_Y_LASER1 (m)	0.1	Q_SONAR	$\left[\begin{array}{cc} 10^{-1} & 0\\ 0 & 10^{-1} \end{array}\right]$	THRESHOLD	0.8
YAW_LASER1 (°)	10	DT_LASER	0.05	SETPOINT_STEP	3
POS_X_LASER2 (m)	0.2	P_LASER	50		

**Table 5:** S&A system test with static vehicle and moving obstacle, number of obstacle positions detected inside/outside the expected interval and relative error, per sensor type.

Sensor	Inside	Outside	Error (%)
Sonar1	28	3	9.7
Sonar2	32	1	3.0
Laser1	27	0	0.0
Laser2	30	0	0.0
Lidar	29	5	14.7

## Data Availability

The data presented in this study are available on request from the corresponding authors. The data are not publicly available due to privacy.

## Abbreviations

The following abbreviations are used in this manuscript:

ADS-B	Automatic Dependent Surveillance-Broadcast
I2C	Inter-Integrated Circuit
TELEM	telemetry
CM4	Computer Module 4
ENU	East-North-Up
ESC	Electronic Speed Controller
FOV	Field of View
GCS	Ground Control Station
GPS	Global Positioning System

HITL	Hardware In the Loop
HUD	head-up display
IMU	Inertial Measurement Unit
LiDAR	Light Detection and Range
MTOW	Maximum Take-Off Weight
RADAR	Radio Detection and Ranging
NED	North-East-Down
RTT	Rapidly exploring Random Tree
ROS	Robotic Operating System
S&A	Sense and Avoid
SITL	Software In the Loop
TCAS	Traffic Alert and Collision Avoidance System
UAV	Unmanned Aerial Vehicle
UGV	unmanned ground vehicle
uORB	micro Object Request Broker
VFH	Vector Field Histogram
